# Post-traumatic epilepsy: bridging pathogenesis, diagnosis, and pharmacotherapeutic strategies

**DOI:** 10.3389/fphar.2025.1697391

**Published:** 2025-11-06

**Authors:** Xiaoyu Yang, Siyang Chen, Haozhou Wang, Tong Sun, Ke Wu

**Affiliations:** 1 Department of Neurosurgery, West China Hospital, West China School of Medicine, Sichuan University, Chengdu, Sichuan, China; 2 Health Management Center, West China School of Public Health and West China Fourth Hospital, Sichuan University, Chengdu, Sichuan, China; 3 Department of Neurosurgery, Xichang Peoples’ Hospital, Liangshan, Sichuan, China

**Keywords:** traumatic brain injury, epilepsy, seizure, predictors, prophylaxis, treatments

## Abstract

Post-traumatic epilepsy (PTE)—affecting 2%–50% of traumatic brain injury (TBI) survivors with severity-dependent incidence—drives secondary neurodegeneration through elevated intracranial pressure (ICP), axonal injury, and neuroinflammatory cascades. While integrated risk stratification (e.g., cortical contusion, acute subdural hematoma on CT; blood-brain barrier disruption via dynamic contrast-enhanced MRI) enhances epileptogenesis prediction, mechanistic understanding of circuit reorganization underlying chronic hyperexcitability remains incomplete. Diagnosis integrates trauma history, seizure semiology, and multimodal monitoring (high-density EEG correlated with [18F] FDG-PET hypometabolism), yet evidence-based prophylaxis is confined to early PTE prevention. In this review, we will describe the progress in incidence, predictors, pathophysiological mechanisms diagnosis, prophylaxis, and treatments with the respect to PTE, providing a comprehensive overview of this subject.

## Introduction

An epileptic seizure is featured as a “transient occurrence of symptoms due to abnormal excessive or synchronous neuronal activity in the brain” (Fisher et al., 2014). Since the seizure frequently occurs in the setting of a potentially epileptogenic brain insult such as traumatic brain injury (TBI), it is used to recognizing the specific form of epilepsy as post-traumatic epilepsy (PTE) (Saletti et al., 2019). However, PTE should be distinguished from repeated seizures caused by metabolic disruption of the brain in the early stage following TBI (Akbar et al., 2024). Although there are various classifications of PTE, the most commonly accepted and widely used classifications by many neurologists based on the timing of the occurrence of PTE are shown as below: (1) immediate seizures, usually defined as occurring within 24 h after TBI; (2) early seizures, usually defined as occurring within 1 week after TBI; and (3) late seizures, defined as occurring over 1 week after TBI (Laing et al., 2022). Studies on hospitalized patients with TBI showed that almost a half of patients with early PTE develop within the first 24 h of injury, which is defined as immediate seizures (Kriukova et al., 2023). Of the seizures that occur within the first 4 weeks of injury, about 10% occur within the first week. Of the late seizures which are characterized by a relatively longer latency, about 40% develop within the first 6 months of injury, 50%–60% develop within the first 12 months of injury, and approximately 80% develop within 2 years of the injury (Ferguson et al., 2010; Sharma et al., 2019). Post-traumatic epilepsy (PTE) develops through multifaceted molecular mechanisms after traumatic brain injury (TBI). Neuroinflammation involving cytokines (e.g., IL-1β, TNF-α) and signaling pathways (e.g., RAGE/TLR4) promotes epileptogenesis (Rawat et al., 2022; Ping et al., 2021). Dysregulation of ion channels, including P2X7 receptor-mediated calcium influx and CaMKII-modulated Nav1.6 hyperactivity, leads to neuronal excitotoxicity and hyperexcitability (Alv et al., 2025; Zybura et al., 2022). Cellular remodeling in the dentate gyrus involves gliosis, synaptic reorganization, and altered gene expression, disrupting neural networks (Gudenschwager-Basso et al., 2023). Oxidative stress exacerbates these processes by inducing mitochondrial dysfunction and neuronal damage (Rawat et al., 2022). These interconnected mechanisms form a complex molecular network underlying PTE.

The search strategy and the flow diagram for included/excluded studies were shown in Supplementary Material. We searched the literature via the following databases: PubMed, Embase, Web of Science, Cochrane Library, and Google Scholar, from establishment to 30 June 2025. We analyzed randomized controlled trials (RCTs), case-control studies, cross-sectional or cohort studies, epidemiologic reports, animal studies, and reviews, that are published in English. Editorials, conference abstracts, case reports, and any non-original studies, are excluded. We used “epilepsy”, “epileptogenesis”, “seizure”, “convulsions”, “posttraumatic epilepsy”, “posttraumatic traumatic seizure”, “brain injury”, “trauma” as the main search terms. The objective of this review is to provide a comprehensive and systematic overview of the epidemiology, pathophysiological mechanisms, diagnosis, and pharmacotherapy of post-traumatic epilepsy.

## Incidence of PTE

Epilepsy remains a common and debilitating complication after TBI. According to the US Center for Disease Control and Prevention (CDC), an estimated 2.5 million Americans suffered a TBI annually, of whom 15% develop post-traumatic early seizures (Lucke-Wold et al., 2015; Zangbar et al., 2016). Various studies reported the incidence of PTE ranging from 2% to as high as 50% that depends on the severity of the injury (Keith and Huang, 2019). Zangbar et al. (Zangbar et al., 2016) analyzed 5551 trauma patients at a US level-one trauma center in a 3-year retrospective study, of whom 1795 were diagnosed with severe TBI, suggesting severe TBI patients were 25 times more likely than the mild TBI patients to develop seizures. In line with the investigation of Zangbar et al., an observational study indicated the incidence was about 2% for mild injuries, 4% for moderate injuries, and over 15% for severe injuries (Annegers et al., 1998). The current data also suggested that PTE was the most prevalent acquired type of epilepsy in young adults (Lucke-Wold et al., 2015). In the terms of cases where the etiology of epilepsy could be identified, trauma is the cause of epilepsy in almost 30% of individuals who develop seizures between ages 15 and 34 years while it is a cause in approximately 15% of children aged 14 years or younger (Piccenna et al., 2017). Post-traumatic epilepsy (PTE) is mainly caused by Traumatic brain injury (TBI), which is an extremely threatening trauma that results in death and disability worldwide (Kotas et al., 2024). Some new findings found that post-traumatic epilepsy (PTE) are contributed to secondary brain damage after TBI and are associated with increased hospital length of stay, mortality, and worse functional outcomes (Pease et al., 2024). In other researcher, Joshua Laing et al. (Gugger and Diaz-Arrastia, 2022) conclude that the role of early posttraumatic seizures (EPS) in the subsequent development of recurrent unprovoked seizures, or posttraumatic epilepsy (PTE), is not well understood. Early posttraumatic seizures may increase the risk of PTE. The authors revealed that EPS may have some influence on PTE, but the specific mechanisms are not well known. The authors also pointed out some risk factors that may contribute to PTE. Pre-injury patient factors such as a history of alcohol misuse and medical comorbidities, previously associated with EPS may interact with TBI and decompensation during acute admission, contributing to EPS separately. Greater TBI severity, measured by surrogate initial GCS or more accurately via composite severity scores such as AIS, confers a worse acute neurological insult, provoking EPS. The cerebral irritation and edema caused by neurosurgical procedures and some in-hospital complications such as sepsis and metabolic derangements, can also increases the risk of EPS (Massimi et al., 2022).

## Predictors of PTE

As summarized before, many researchers adopted a set of definitions with regard to PTE according the timing of the occurrence of seizures: (1) immediate seizures; (2) early seizures; (3) late seizures (Appavu et al., 2022). The incidence of epilepsy after different brain insults were reported before, where PTE has an increased risk of incidence rate compared to the general population and followed by subarachnoid hemorrhage and brain tumor (Herman, 2002). It was widely accepted that the risk factors for epilepsy after brain injury is strongly associated with the severity of TBI, with a 4-fold risk after moderate TBI and 1.5-fold risk after mild TBI (Piccenna et al., 2017).

Numerous studies have reported the risk factors of PTE. A systematic review and meta-analysis was conducted to identify potential factors and the probability for PTE include male patients (RR = 1.32), alcohol abuse (RR = 2.18), post-traumatic amnesia (RR = 1.31), focal neurologic signs (RR = 1.42), and consciousness loss at initial head injury (RR = 1.62) (Spencer et al., 2019). The risk of PTE was significantly higher for those patients with immediate seizures (RR = 5.14). Moreover, the brain image showed that skull fracture (RR = 2.27), midline shift (RR = 1.46), cerebral contusion (RR = 2.35), subarachnoid hemorrhage (RR = 2.00), and intracranial hemorrhage (RR = 2.65) were related to a higher incidence of developing seizures. M. Pease et al. (Pease et al., 2022) performed a retrospective analysis of a prospective database including consecutive patients with severe traumatic brain injury (TBI) treated at a single Level 1 trauma center from 2002 through 2018. In this cohort study, the researchers confirmed several risk factors including DHC, age, and CNS infection for the first late PTE and male gender and shunt independence for PTE recurrence. Relating to previous research, there are some markers of injury severity affiliated with PTE such as penetrating injury, depressed skull fractures, and cerebral contusions (Kumar et al., 2019). The results shows that due to the increased incidence of PTE and several reliable risk factors, the utilization of severe TBI should be applied to establish models of epileptigenesis and screen for antiepileptic therapy.

Furthermore, a closer look at the risk factors for early and late seizures. Risk factors for early seizures among children include age (especially less than 5 years old), acute intracerebral hematoma, acute subdural hematoma, diffuse cerebral edema, retention of intracranial metal fragments, focal neurological deficit, skull fracture, loss of consciousness, posttraumatic amnesia more than 30 min (Keret et al., 2017). Another study published by Englander et al. showed that the risk factors for late seizures were biparietal contusions, dura penetration with bone and metal fragments, multiple intracranial surgeries, multiple subcortical contusions, subdural hematoma, midline shift over 5 mm, and multiple cerebral contusions. And the study reported that there is an increasing risk of late seizures for patients with multiple brain contusions compared to a single cerebral contusion (Englander et al., 2003).

Moreover, a new study published by Surina Fordingtan (Fordington et al., 2020) revealed some new risk factors regarding PTE. Surina Fordington et al. conducted a review about the basic information of post-traumatic epilepsy (PTE). In this review, the researchers analyzed some factors that may result in post-traumatic epilepsy. The researches explained that following injury there are a number of processes, including necrosis, microhaemorrhage, axonal injury, apoptosis, demyelination, microgliosis, inflammation and oxidative stress and later phases of neurodegeneration, regeneration, revascularisation and remodeling which may contribute to the circuit changes resulting in later epilepsy. He also pointed out some imaging markers such as gradient echo MRI for blood products or diffusion tensor MRI for pathway remodeling, evidence of thalamic or hippocampal damage may help to stratify risk.

## Mechanisms of PTE

**FIGURE 1:**
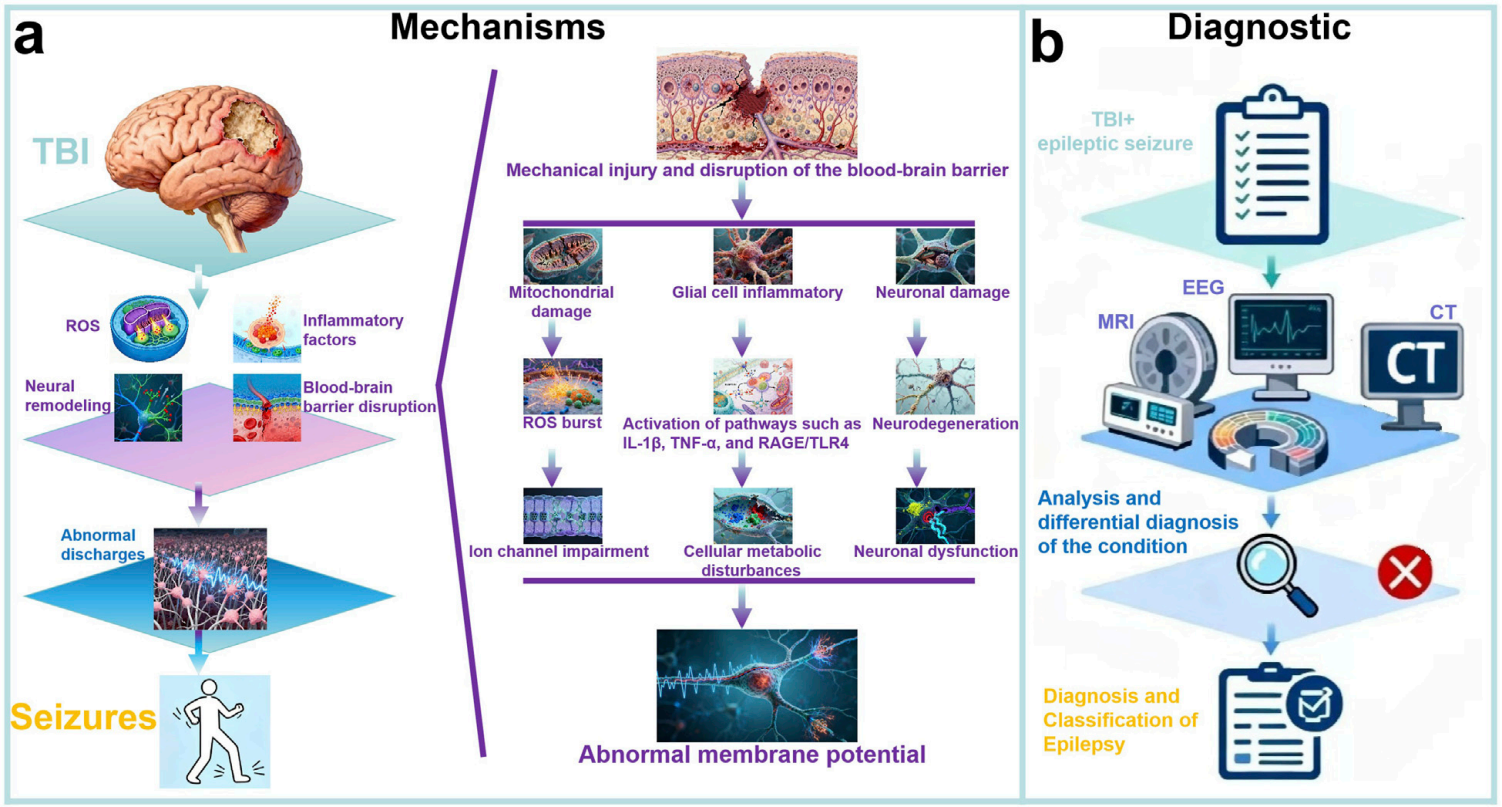
Mechanisms and Diagnostic Process of PTE. **(a)** Mechanisms of PTE: PTE develops after TBI through interconnected molecular mechanisms. Neuroinflammation (e.g., IL-1β, TNF-α, RAGE/TLR4 pathways) promotes epileptogenesis, exacerbated by blood-brain barrier disruption. Cellular remodeling in the dentate gyrus impairs neural networks, while oxidative stress induces mitochondrial dysfunction. These processes form a complex molecular network underlying PTE; **(b)** Diagnostic of PTE: The diagnosis and classification of PTE are confirmed by integrating TBI and seizure history, examinations such as EEG, CT, and MRI, as well as history analysis and differential diagnosis of epilepsy.

We summarized the Mechanisms of PTE in Figure 1a. Post-traumatic epilepsy (PTE), as a chronic condition secondary to traumatic brain injury (TBI), involves complex multi-level molecular biological mechanisms in its pathogenesis and progression (Aykin et al., 2025). Regarding inflammatory response mechanisms, TBI triggers a series of pathological processes including innate immune system activation, release of inflammatory factors, and oxidative stress, which directly promote epileptogenesis (Kajevu et al., 2025). Specifically, brain injury leads to features such as widespread inflammatory responses, blood-brain barrier disruption, and oxidative stress, and these excitatory characteristics may ultimately contribute to the development of PTE (Rawat et al., 2022). The inflammatory response involves the release of pro-inflammatory cytokines (e.g., IL-1β, TNF-α), which enhance neuronal excitability and create a microenvironment conducive to seizure generation. In terms of specific signaling pathways, the receptor for advanced glycation end products (RAGE) and Toll-like receptor 4 (TLR4) signaling pathways have been demonstrated to play critical roles; these pathways promote long-term epileptogenesis by mediating neuroinflammation and glial cell activation, and blocking RAGE or TLR4 signaling is considered an effective strategy for preventing PTE (Ping et al., 2021).

At the level of receptor and ion channel mechanisms, dysregulation of ion channels and receptors at the molecular level constitutes the core pathological basis of PTE. Among these, the P2X7 receptor (P2X7R), an ATP-dependent cationic membrane channel, plays a significant role in post-traumatic epileptogenesis. This receptor is widely distributed throughout the brain, and its activation can lead to calcium influx and excitotoxicity, thereby promoting neuronal death and subsequent epileptic seizures. The molecular mechanisms involved include imbalance in synaptic transmission and amplification of inflammation (Alv et al., 2025). Simultaneously, gain-of-function changes in sodium channels (such as Nav1.6) are closely associated with epileptogenesis. This process involves regulation by calcium/calmodulin-dependent protein kinase II (CaMKII), which modulates Nav1.6 activity through post-translational modifications, leading to neuronal hyperexcitability. Although this mechanism is prevalent in epilepsy generally, its specific contribution to PTE in the context of TBI can be elucidated through model studies (Zybura et al., 2022).

In terms of cellular remodeling and molecular changes, molecular cascades and abnormal cellular remodeling in specific brain regions following TBI collectively drive epileptogenesis (Golub and Reddy, 2022a). The dentate gyrus, identified as a key region highly susceptible to injury, may experience epileptogenesis due to abnormal cellular remodeling events (including gliosis and synaptic reorganization) and critical molecular changes (such as increased astrocyte expression) (Gudenschwager-Basso et al., 2023). RNA sequencing (RNA-Seq) analyses have revealed differential gene expression in animal models of PTE, involving dysregulation of glial cell activation and signaling molecules (such as inflammatory markers). These changes manifest specifically as neuronal loss, altered synaptic plasticity, and abnormal expression of related proteins such as cell adhesion molecules and growth factors, ultimately leading to disrupted neural network connectivity and spontaneous epileptic seizures (Gudenschwager-Basso et al., 2023).

Furthermore, oxidative stress and free radical damage, as secondary injury characteristics of TBI, promote cell death and increased excitability by inducing mitochondrial dysfunction and DNA damage, thereby amplifying the aforementioned molecular mechanisms and advancing the pathological progression of PTE (Rawat et al., 2022). These mechanisms are interconnected and together constitute a complex molecular network underlying epileptogenesis following TBI.

## Diagnosis of PTE

According to the recent clinical definition of epilepsy proposed by The International League Against Epilepsy (ILAE), epilepsy is a neurological syndrome defined by any of the following conditions: “(1) at least two unprovoked seizures occurring over 24 h apart; (2) one single unprovoked seizures and a probability of further seizures similar to the general recurrence risk (at least 60%) after two unprovoked seizures occurring of seizures that is accompanied by a recurrence rate of more than 60%; (3) an epilepsy syndrome” (Fisher et al., 2014).

We summarized the diagnostic process for PTE in Figure 1b. Considering about the different severity, injury mechanism, and location of TBI, the clinical manifestations of PTE might be various. However, PTE is sometimes well recognized in clinical practice (Thijs et al., 2019). The diagnosis of PTE actually did not have a definite or gold standard, and radically depends on the clinical manifestations and electroencephalography (EEG) examination, particularly the type of seizures and the timeline to develop seizures after injury (immediate seizures, early seizures, or late seizures) (Moshé et al., 2015). A detailed description, or a reliable eyewitness, or video, possibly provides the best evidence (Amin et al., 2021). Sometimes, the diagnosis of PTE is relatively difficult since the symptoms of epilepsy are non-specific and patients could be accompanied with systematic diseases such as convulsive syncope, cardiogenic attacks, or consciousness disorders, where continuous EEG monitoring (usually 24 h) is essential to recognize seizures (Wilson et al., 2017; Vázquez-Sánchez et al., 2021). Seizures with typical convulsive features are more likely to be clinically diagnosed, however, the non-convulsive seizures that only be detected by continuous EEG monitoring should not be neglected since they are usually associated with poor outcomes. In this light, physicians must exclude some events that are related to other diseases and primary epilepsy (Karki et al., 2024). James J. Gugger et al. (Zimmermann et al., 2016) summarized a series of previous researches and generalized some common diagnostic methods for post-traumatic epilepsy. The researchers concluded that Early posttraumatic seizures generally manifest within the initial 24 h following an injury and exhibit considerable clinical heterogeneity, varying from entirely subclinical presentations to convulsive episodes. Usually, seizures within the first 24 h after injury are categorized as immediate posttraumatic seizures. With the routine implementation of continuous electroencephalography (cEEG), the incidence of epileptic seizures is significantly elevated (Hanin et al., 2021). Furthermore, early studies that relied solely on clinical semiology for seizure identification may only represent a fraction of the actual prevalence (Kukuruzović et al., 2021). But with the help of combined scalp and intracranial EEG, it can show even higher rates of electrographic seizures than seen with scalp EEG alone (Vespa et al., 2016; Varatharajah et al., 2021).

Importantly, lumbar puncture and neuroimage including Computed Tomography (CT) and Magnetic Resonance Imaging (MRI) are necessary in some cases, which are capable of detecting the PTE and predicting the occurrence of seizures (Garner et al., 2019). In recent researches, the researchers concluded that although seizures may not be clinically apparent, invasive monitoring has demonstrated that electrographic seizures are associated with intracranial hypertension and brain tissue hypoxia (Arslan et al., 2022). The other researches also concluded that early posttraumatic seizures are linked with subsequent hippocampal atrophy and PTE, although at a level of risk that is in keeping with acute symptomatic seizures (Tubi et al., 2019).

## Pharmacotherapy of PTE

### Early and late prophylaxis

Seizure prophylaxis is central to the management of patients with TBI. A summary of prophylaxis of PTE based on some clinical trials has been shown in Supplementary 1. A comparative analysis of commonly used drugs in PTE is shown in Table 1. It has long been known that the use of antiepileptic drugs (AEDs) could significantly decrease the incidence of early PTE occurring within 7 days of injury despite the potential side effects related to its use, which remain a serious problem in all neurosurgical patients with data for brain tumor patients alone reporting severe side effects in up to 23.8% of patients (Zafar et al., 2012; Samara et al., 2023). Although there are diversified results with regards to the benefits and complications of the seizure prophylaxis, both guideline recommendations (The Brain Trauma Foundation Guidelines for the Management of Severe Traumatic Brain Injury, Fourth Edition) and previous literature suggest prophylactic use of AED is recommended for preventing early PTE (Carney et al., 2017).

**TABLE 1:** Comparative analysis of commonly used drugs in PTE.

Drug name	Phenytoin	Levetiracetam	Valproate	Carbamazepine	Lamotrigine	Zonisamide
Application level in PTE	Guideline-recommended (classic choice)	First-line/Preferred (clinical practice trend)	Effective alternative option	Non-first-line medication	Non-first-line medication	Non-first-line medication
Mechanism	Sodium channel blocker: inhibits high-frequency repetitive neuronal firing	Binds to synaptic vesicle protein SV2A, modulating neurotransmitter release	Multiple mechanisms: enhances GABAergic inhibition, blocks sodium channels and T-type calcium channels	Sodium channel blocker	Sodium channel blocker, inhibits glutamate release	Multiple mechanisms: blocks sodium channels and T-type calcium channels
Advantages	Widely used, low cost	Excellent safety profile,few drug interactions, no routine monitoring required	Broad-spectrum antiepileptic activity	Well-established efficacy for focal seizures	Well-tolerated, first-line for focal and generalized seizures	Once-daily dosing with high compliance
Disadvantages	Frequent/severe side effects; small dose changes cause large blood level swings; many drug interactions due to enzyme induction	Main side effects: drowsiness, fatigue, mood changes. Intravenous form may be costly	Major risks: high teratogenicity, weight gain, tremors, hair loss, thrombocytopenia, hepatotoxicity; frequent drug interactions	Metabolism self-induces (levels drop weeks later, needs dose adjustment); Serious side effects: hyponatremia, rash, aplastic anemia; Strong enzyme inducer—multiple drug interactions	Slow titration required (due to SJS rash risk)—not for acute use; Metabolism affected by valproic acid	Side effects include​ kidney stones, sedation, weight loss, reduced sweating (caution in children); Limited data for PTE.
Pharmacokinetic characteristics	Requires blood level monitoring; high protein binding increases free fraction in hypoalbuminemia	Renal excretion—dose adjustment needed in renal impairment; Wide therapeutic window—no routine monitoring needed	Requires therapeutic drug monitoring; hepatic enzyme inhibitor with drug interaction potential	Requires therapeutic drug monitoring; complex pharmacokinetics and side effects limit its use for acute prophylaxis	Routine therapeutic drug monitoring is not required; complex titration limits acute-phase use	Partially metabolized by the liver; no routine monitoring required
Strength of evidence	Strong	Strong	Moderate	Moderate	Weak	Very weak

In TBI, phenytoin remains the most commonly used and widely studied AED but the efficacy and safety of phenytoin medications are not as expected owing to the numerous side effects such as dystrophy, neurological disorders, and drug interactions (Wat et al., 2019; Arora et al., 2021). Moreover, patients should be periodically monitored therapeutic levels in serum to ensure the efficiency ascribing to the possibly disproportionate changes following small changes in drug dosage and metabolism (Prasertsup et al., 2023). Other widely used AEDs, including valproate, carbamazepine, and levetiracetam, emerge as novel, effective, and alternative treatments with an increasing number of research interests (Lou and Cui, 2022). Since the application of various AEDs in clinic, a great deal of attention has been directly given to the comparison of safety and effectiveness among four forementioned AEDs, raising a question that the best option for the prophylaxis of PTE. Ma et al. (2010) conducted a retrospective study from a single institution at the Department of Neurosurgery, Nanjing General Hospital of Nanjing Command to determine the safety and effectiveness of valproate in seizure prophylaxis following TBI. In this 2-year study, a total of 159 patients with TBI were included, of whom 42 patients received administration of sodium valproate, and the results showed prophylactic use of valproate was associated with a lower incidence of PTE since seven patients (4.4%) in the control group developed seizures while valproate-treated patients did not develop seizures but without statistical significance. Beghi (2003) conducted a Cochrane systematic review of 890 patients from 10 randomized clinical trials (RCTs) to demonstrate and compare the efficacy of the prophylactic use of phenytoin and carbamazepine, indicating equal efficacy in reducing the risk of early PTE while a higher incidence of adverse events was observed among phenytoin users Levetiracetam is a more recent AED with some favorable characteristics including avoidance of cognitive deficit, improvement in neuropsychological functions, safety in pregnancy, wide therapeutic window with predictable pharmacokinetics, and unnecessary of serum monitoring (Bakr and Belli, 2018). A randomized controlled trial conducted by Szaflarski et al. (2010) suggested better long-term outcomes for levetiracetam-treated patients compared to Phenytoin. Specifically, it was a prospective, single-center, randomized, single-blinded comparative trial to compare the safety and effectiveness of phenytoin with those of levetiracetam at the ratio of 2:1 and recruited a total of 52 participants with severe TBI or subarachnoid hemorrhage, of whom 34 patients received levetiracetam treatments and 18 patients received phenytoin treatments. A lower Disability Rating Scale score at 3 months (*p* = 0.042) and a higher Glasgow Outcomes Scale score at 6 months (*p* = 0.039) were observed in the levetiracetam group compared to phenytoin. Levetiracetam-treated patients showed a lower frequency of worsened neurological status (*p* = 0.024), and gastrointestinal problems (*p* = 0.043) comparing to phenytoin. Both continuous EEG (cEEG) (14.7% vs. 16.6%; *p* = 1.0) for 72 h and EEG 6 months (5.0% vs. 0.0%; *p* = 1.0) showed no statistical differences between groups. Similarly, the incidence of seizures did not differ between the two groups. In accordance with the work of Szaflarski et al., a retrospective observational study suggested a significant shift toward the prescribing of levetiracetam over phenytoin for the prevention of seizures after TBI, despite the lack of insufficient evidence (Kruer et al., 2013). A subsequent systematic review conducted by Zafar et al. (2012) to analyze the relative effectiveness of two AEDs suggested levetiracetam and phenytoin had equal efficacy in seizure prophylaxis following TBI. Fisher, (2023) conducted a review regarding the efficacy of deep brain stimulation (DBS) in the treatment of refractory epilepsy. DBS for epilepsy has been targeted to the anterior nucleus (ANT), which is the only target that is approved by FDA. The research revealed that symmetrical stimulation of ANT reduced seizures by 40.5% at 3 months in the controlled phase (p = 0.038) and 75% by 5 years in the uncontrolled phase. There are also various of side effects such as paresthesia, acute hemorrhage, infection, occasional increased seizures, and usually transient effects on mood and memory. Tahir Hakama et al. (Hakami, 2021) systematically studied the usage of AEDs in the primary therapy for epilepsy. In his review, he concluded that different types of AEDs are suitable for different seizure type. Several AEDs are useful as first‐line monotherapy in focal seizures, including lamotrigine, carbamazepine, phenytoin, levetiracetam, and zonisamide. Valproate remains the first‐line drug for many patients with generalized and unclassified epilepsies. But he also indicated that women in childbearing are prohibited from taking valproate because of teratogenicity. Harison Gopalan and P, (2023) designed a questionnaire consisting of sixteen questions with the help of Google and transmit to Neurologists and Neurosurgeons around the world, aiming to assess the variability of management practices of PTE and PTE and highlight the pressing need to formulate uniform practice guidelines. The researchers received 220 responses and concluded that the majority of Neurologists and Neurosurgeons around the world would start an AED prophylaxis to prevent PTE. The researchers also pointed out that Phenytoin (n = 98; 48.5%) followed by Levetiracetam (n = 78; 38.6%) was the preferred drug, although the latter was significantly preferred by high and upper middle-income countries (*p <* 0*.*001).

Although there is a common acceptance for the prevention of early PTE, the benefits of seizure prophylaxis for late PTE remain controversial (Pease et al., 2022). A recently reported meta-analysis to quantify the association between the prophylactic use of phenytoin or more recent levetiracetam and risk of late seizures after TBI analyzed four studies that compared phenytoin with placebo and two studies that compared levetiracetam with placebo with regard to the prevention of late PTE, and demonstrated no significant difference in the prevention of late PTE between patients who received phenytoin or levetiracetam and patients who received placebo (Wilso et al., 2018). Simultaneously, a few studies on the prevention of late PTE showed variable success with a significant reduction in the incidence of late PTE, providing modest evidence as a result of relatively small size and open-label design (Wat et al., 2019; Bakr and Belli, 2018). At present, there is lack of evidence to recommend AEDs in the prevention of late PTE (Golub and Reddy, 2022b).

### Long-term treatments of PTE

In current clinical practice, the prevention and treatment of PTE mainly include the following strategies: (1) after the occurrence of severe TBI, take preventive measures to prevent the occurrence of early PTE in time; (2) when late PTE occurs, take first‐line AEDs that are effective for focal epilepsy; (3) for patients with drug‐resistant PTE or unable to tolerate drug side effects, surgical treatment can be selected (Zimmermann et al., 2017; Pingue et al., 2021).

Head injury frequently leads to PTE, resulting in worse outcomes for patients with TBI. The occurrence of PTE might elevate the intracranial pressure (ICP), damage white matter, impair brain development and cause neurodegeneration (Baye et al., 2024). Severely, status seizures could cause acute cardiac and respiratory failure, brainstem herniation, or death. Additionally, late PTE has a negative impact on quality of life, return to work, and driving (Mee et al., 2019). It is therefore essential to start anti-epileptic treatments once a patient develops a seizure. Even though AEDs are associated with numerous side effects, they remain the mainstay of treatment for patients with PTE since the overall benefit is thought to outweigh the known complications related to such treatment (Fordington et al., 2020). The most commonly used AEDs include phenytoin, levetiracetam, valproate, and carbamazepine. (Dehury et al., 2023). Investigators in previous studies have evaluated the benefits of the use of AEDs alone, or in combination, but the current evidence did not reveal a significant difference among pharmacological treatments (Mee et al., 2019). Interestingly, levetiracetam is the preferred drug according to a recent survey of US clinicians (Szaflarski, 2015), and a similar trend has also been demonstrated in Europe (Huijben et al., 2018). In the other aspect, the researches also focus on regulating glutamate homeostasis to refrain neuronal death and neural network remodeling. In a study conducted by Gao et al. (2023), the researchers listed some drug which can regulate glutamate homeostasis, this in turn inhibits the neuroexcitatory toxins caused by glutamate, therefore refraining neuronal death and neural network remodeling. There is a latent period between brain injury and PTE, a series of molecular biological changes caused by tissue injury, which provides a window for drug intervention in preventing late epilepsy. In this latent period, many relevant treatments can be executed to prevent the injuries from post-traumatic epilepsy, including a variety of AEDs, which may also bring numerous side effects. All in all, the mechanism of PTE is complex, involving multiple causes such as neuroinflammation, oxidative stress, and excitotoxicity. (Gao et al., 2023). Various aspects of research are needed for the long-term treatment of post-traumatic epilepsy.

On the other hand, the optimal duration of treatment for this group of patients is unclear to date. In this regard, many studies have been conducted regarding the subject, but few were RCTs. No matter how long the AEDs mediations last, it is rather certain that patients should be periodically monitored therapeutic levels in serum, together along with the routine or 24-h EEG examination, to ensure the therapeutic dose, safety, and efficiency (Klimpel et al., 2021).

## Perspective

PTE progression bridges neuroinflammatory-axonal-circuit pathologies with precision risk stratification and evidence-based management, providing an integrative framework for translational research. Current evidence demonstrates that integrated risk stratification models—incorporating established clinical factors (e.g., acute subdural hematoma, cortical contusion) and advanced neuroimaging biomarkers (e.g., blood-brain barrier permeability on dynamic contrast-enhanced MRI, iron deposition via quantitative susceptibility mapping)—enable probabilistic forecasting of epileptogenesis (Kumar et al., 2024; Na et al., 2022). Despite diagnostic reliance on trauma history, seizure semiology, and multimodal monitoring, fundamental gaps persist in delineating the spatiotemporal evolution of post-TBI circuitopathies. Guideline-directed prophylaxis supports AEDs for early PTE prevention, yet late PTE remains therapeutically unaddressed due to insufficient efficacy data. Critical analysis reveals divergent pharmacotherapeutic profiles: while meta-analyses indicate comparable seizure control among LEV, VPA, and phenytoin (PHT), PHT exhibits significantly higher neurotoxicity, whereas LEV/VPA demonstrate superior target specificity—LEV modulates SV2A-mediated synaptic vesicle cycling without cytochrome P450 induction, and VPA promotes histone deacetylase-dependent neuroprotection. This therapeutic dichotomy necessitates: (1) prospective trials evaluating disease-modifying agents; (2) pharmacogenomic strategies to mitigate PHT toxicity; and (3) validation of fluid biomarkers as surrogate endpoints for long-term neuroprotection assessment.
