# Supplementary material for: Post-traumatic epilepsy: bridging pathogenesis, diagnosis, and pharmacotherapeutic strategies
Source: Front Pharmacol. 2025 Nov 6;16:1697391. doi: 10.3389/fphar.2025.1697391 (PMC12630123; doi:10.3389/fphar.2025.1697391)
Supplement: Supplementary file 1 [file Supplementaryfile1.docx]

**Supplementary Table 1** Summary of the studies on the prophylaxis of post-traumatic epilepsy

| Author & Year | Study design | Treatment | Dose | Study group  n (N) | Control group  n (N) |
| --- | --- | --- | --- | --- | --- |
| Early prophylaxis | | | | | |
| Young et al.,[^1^](#_ENREF_1)1983 | RCT | PHE vs Placebo | 11-13 mg/kg/d + 8.8 mg/kg/d | 5 (136) | 4 (108) |
| Temkin et al., [^2^](#_ENREF_2) 1990 | RCT | PHE vs Placebo | 20 mg/kg/d + 200-1200 mg/d | 7 (208)* | 26 (196) |
| Ohimor et al., [^3^](#_ENREF_3) 1996 | Retrospective | PHE vs No prophylaxis | Not available | 0 (44)* | 9 (41) |
| Young et al., [^4^](#_ENREF_4) 2004 | RCT | PHE vs Placebo | 18 mg/kg/d + 6 mg/kg/d | 3 (46) | 3 (56) |
| Jones et al.,[^5^](#_ENREF_5) 2008 | Retrospective & Prospective | PHE vs LEV | PHE: Not available  LEV: 500 mg/12 h | 0 (41) | 1 (32) |
| Ma et al.,[^6^](#_ENREF_6)  2010 | Retrospective | VAL vs No prophylaxis | 10-15 mg/kg/d | 0 (35)* | 7 (124) |
| Debenham et al.,[^7^](#_ENREF_7) 2011 | Retrospective | PHE vs No prophylaxis | 17 mg/kg/d + 100 mg/8 h | 23 (653)* | 31 (355) |
| Liesemer et al.,[^8^](#_ENREF_8) 2011 | Retrospective | PHE vs Placebo | Not available | 3 (132)* | 26 (116) |
| Inaba at al.,[^9^](#_ENREF_9) 2013 | Prospective | PHE vs LEV | PHE: 15 mg/kg+4.5 mg/kg/d  LEV: 1000 mg/12 h | 6 (407) | 6 (406) |
| Gabriel et al.,[^10^](#_ENREF_10) 2014 | Prospective | PHE vs LEV | PHE: 20 mg/kg/d +100 mg/8 h  LEV: 500 mg/12 h | 3 (14) | 0 (5) |
| Bhullar et al.,[^11^](#_ENREF_11) 2014 | Retrospective | PHE vs No prophylaxis | 20 mg/kg/d +5 mg/kg/d | 2 (50) | 1 (43) |
| Zangbar et al.,[^12^](#_ENREF_12) 2016 | Retrospective | LEV vs No prophylaxis | Not available | 4 (208) | 7 (208) |
| Late prophylaxis | | | | | |
| Wohns et al.,[^13^](#_ENREF_13) 1979 | Retrospective | PHE vs Placebo | 400 mg/d | 5 (50)* | 6 (12) |
| McQueen et al.,[^14^](#_ENREF_14) 1983 | RCT | PHE vs Placebo | 100 mg/d + 300 mg/d | 8 (84) | 7 (80) |
| Temkin et al.,[^2^](#_ENREF_2) 1990 | RCT | PHE vs Placebo | 20 mg/kg/d + 200-1200 mg/d | 57 (208) | 41 (196) |
| Pechadre et al.,[^15^](#_ENREF_15) 1991 | RCT | PHE vs Placebo | Not available | 2 (34)* | 22 (52) |
| Pearl et al.,[^16^](#_ENREF_16) 2013 | RCT | LEV vs Placebo | 55 mg/kg/d, b.i.d. | 1 (19) | 0 (19) |
| Klein et al.,[^17^](#_ENREF_17) 2012 | RCT | LEV vs Placebo | 55 mg/kg/d, b.i.d. | 6 (66) | 8 (60) |

LEV, levetiracetam; PHE, phenytoin; VAL, valproate; RCT, randomized controlled trial. * *P* <0.05 versus control group.

**References**

1. Young B, Rapp RP, Norton JA, Haack D, Walsh JW. Failure of Prophylactically Administered Phenytoin to Prevent Post-Traumatic Seizures in Children. *Childs Brain.* 1983;10(3): 185-192.

2. Temkin NR, Dikmen SS, Wilensky AJ, Keihm J, Chabal S, Winn HR. A Randomized, Double-Blind Study of Phenytoin for the Prevention of Post-Traumatic Seizures. *New England Journal of Medicine.* 1990;323(8): 497-502. https://doi.org/10.1056/NEJM199008233230801.

3. Ohimor SO, Falcone RE. Phenytoin Prophylaxis in Posttraumatic Head Injury. *Journal of Pharmacy Technology.* 1996;12(4): 160-164. https://doi.org/10.1177/875512259601200410.

4. Young KD, Okada PJ, Sokolove PE, Palchak MJ, Lewis RJ. A Randomized, Double-Blinded, Placebo-Controlled Trial of Phenytoin for the Prevention of Early Posttraumatic Seizures in Children with Moderate to Severe Blunt Head Injury. *Annals of Emergency Medicine.* 2004;43(4): 435-446.

5. Jones KE, Puccio AM, Harshman KJ, et al. Levetiracetam versus phenytoin for seizure prophylaxis in severe traumatic brain injury. *Neurosurg Focus.* 2008;25(4): E3-E3. https://doi.org/10.3171/FOC.2008.25.10.E3.

6. Ma C-y, Xue Y-j, Li M, Zhang Y, Li G-z. Sodium valproate for prevention of early posttraumatic seizures. *Chinese Journal of Traumatology (English Edition).* 2010;13(5): 293-296. https://doi.org/https://doi.org/10.3760/cma.j.issn.1008-1275.2010.05.008.

7. Debenham S, Sabit B, Saluja RS, et al. A critical look at phenytoin use for early post-traumatic seizure prophylaxis. *Canadian Journal of Neurological Sciences.* 2011;38(06): 896-901.

8. Liesemer K, Bratton SL, Zebrack CM, Brockmeyer D, Statler KD. Early Post-Traumatic Seizures in Moderate to Severe Pediatric Traumatic Brain Injury: Rates, Risk Factors, and Clinical Features. *Journal of Neurotrauma.* 2011;28(5): 755-762. https://doi.org/10.1089/neu.2010.1518.

9. Inaba K, Menaker J, Branco BC, et al. A prospective multicenter comparison of levetiracetam versus phenytoin for early posttraumatic seizure prophylaxis. *Journal of Trauma and Acute Care Surgery.* 2013;74(3).

10. Gabriel WM, Rowe AS. Long-Term Comparison of GOS-E Scores in Patients Treated With Phenytoin or Levetiracetam for Posttraumatic Seizure Prophylaxis After Traumatic Brain Injury. *Annals of Pharmacotherapy.* 2014;48(11): 1440-1444. https://doi.org/10.1177/1060028014549013.

11. Bhullar IS, Johnson D, Paul JP, Kerwin AJ, Tepas JJ, III, Frykberg ER. More harm than good: Antiseizure prophylaxis after traumatic brain injury does not decrease seizure rates but may inhibit functional recovery. *Journal of Trauma and Acute Care Surgery.* 2014;76(1).

12. Zangbar B, Khalil M, Gruessner A, et al. Levetiracetam Prophylaxis for Post-traumatic Brain Injury Seizures is Ineffective: A Propensity Score Analysis. *World journal of surgery.* 2016;40(11): 2667-2672. https://doi.org/10.1007/s00268-016-3606-y.

13. Wohns RNW, Wyler AR. Prophylactic phenytoin in severe head injuries. *Journal of neurosurgery.* 1979;51(4): 507-509. https://doi.org/10.3171/jns.1979.51.4.0507.

14. McQueen JK, Blackwood DH, Harris P, Kalbag RM, Johnson AL. Low risk of late post-traumatic seizures following severe head injury: implications for clinical trials of prophylaxis. *J Neurol Neurosurg Psychiatry.* 1983;46(10): 899-904. https://doi.org/10.1136/jnnp.46.10.899.

15. Pechadre JC, Lauxerois M, Colnet G, Commun C, Ch Ab Annes J. [Prevention of late post-traumatic epilepsy by phenytoin in severe brain injuries. 2 years' follow-up]. *Presse Médicale.* 1991;20(18): 841-845.

16. Pearl PL, McCarter R, McGavin CL, et al. Results of phase II levetiracetam trial following acute head injury in children at risk for posttraumatic epilepsy. *Epilepsia.* 2013;54(9): e135-e137. https://doi.org/10.1111/epi.12326.

17. Klein P, Herr D, Pearl PL, et al. Results of phase 2 safety and feasibility study of treatment with levetiracetam for prevention of posttraumatic epilepsy. *Archives of neurology.* 2012;69(10): 1290-1295. https://doi.org/10.1001/archneurol.2012.445.

**Search Strategy**

**Objective:** To identify and retrieve relevant peer-reviewed literature exploring the pathophysiology, diagnostic methods, and pharmacological treatment strategies for post-traumatic epilepsy.

**1. Core Concepts and Keywords**​

The search will be built around three core conceptual groups, with synonyms and related terms identified for each.

**Concept 1**: Post-Traumatic Epilepsy

**Concept 2**: Pathogenesis / Mechanisms

**Concept 3**: Diagnosis & Pharmacotherapy

**2. Database-Specific Search Syntax (PubMed Example)​**​

The following search strings use Boolean operators (AND, OR), truncation (*), and phrase searching ("...").

**Step 1:** Define Search Terms for Each Concept​

**Concept 1:** Post-Traumatic Epilepsy​

Keywords: "Post-Traumatic Epilepsy", "Posttraumatic Epilepsy", "Traumatic Epilepsy", "Post-Traumatic Seizures", "Seizures, Post- Traumatic",

MeSH Terms: Epilepsy, Post-Traumatic, Seizures, Post-Traumatic, Brain Injuries, Traumatic/complications

**Concept 2:** Pathogenesis / Mechanisms​

Keywords: pathogenesis, pathophysiology, mechanism*, etiology*, pathologenesis, neurobiology, epileptogenesis

MeSH Terms: Pathogenesis, Pathophysiology, Etiology, Disease Progression

**Concept 3:** Diagnosis & Pharmacotherapy​

Keywords: diagnosis*, detection, predict*, biomarker, electroencephalography, EEG, MRI, imaging, pharmacotherapy, "drug therapy", antiepileptic, anticonvulsant, treatment, management, prevention, prophylaxis, therapeutics

MeSH Terms: Diagnosis, Biomarkers, Electroencephalography, Magnetic Resonance Imaging, Drug Therapy, Anticonvulsants/therapeutic use, Treatment Outcome, Disease Management

**Step 2:** Combine Terms Within Each Concept (Using OR)​​

**Step 3:** Combine Concepts (Using AND)​​

**3. Inclusion/Exclusion Criteria**​​

**Inclusion:** Human studies; animal models (for pathogenesis); randomized controlled trials, cohort studies, case-control studies, systematic reviews; published in English; published from 2003-2023 (last 20 years, adjustable).

**Exclusion:** Case reports; editorials; non-English papers; studies on non-traumatic epilepsy (e.g., stroke, tumor).

**4. Search Execution and Management​**

a. Execute the search in selected databases.

b. Import all results into a reference manager .

c. Remove duplicate records.

d. Screen titles and abstracts against inclusion/exclusion criteria.

e. Obtain and assess the full text of relevant articles for final inclusion.

f. This strategy ensures a comprehensive and focused retrieval of literature central to the review's objectives.

**
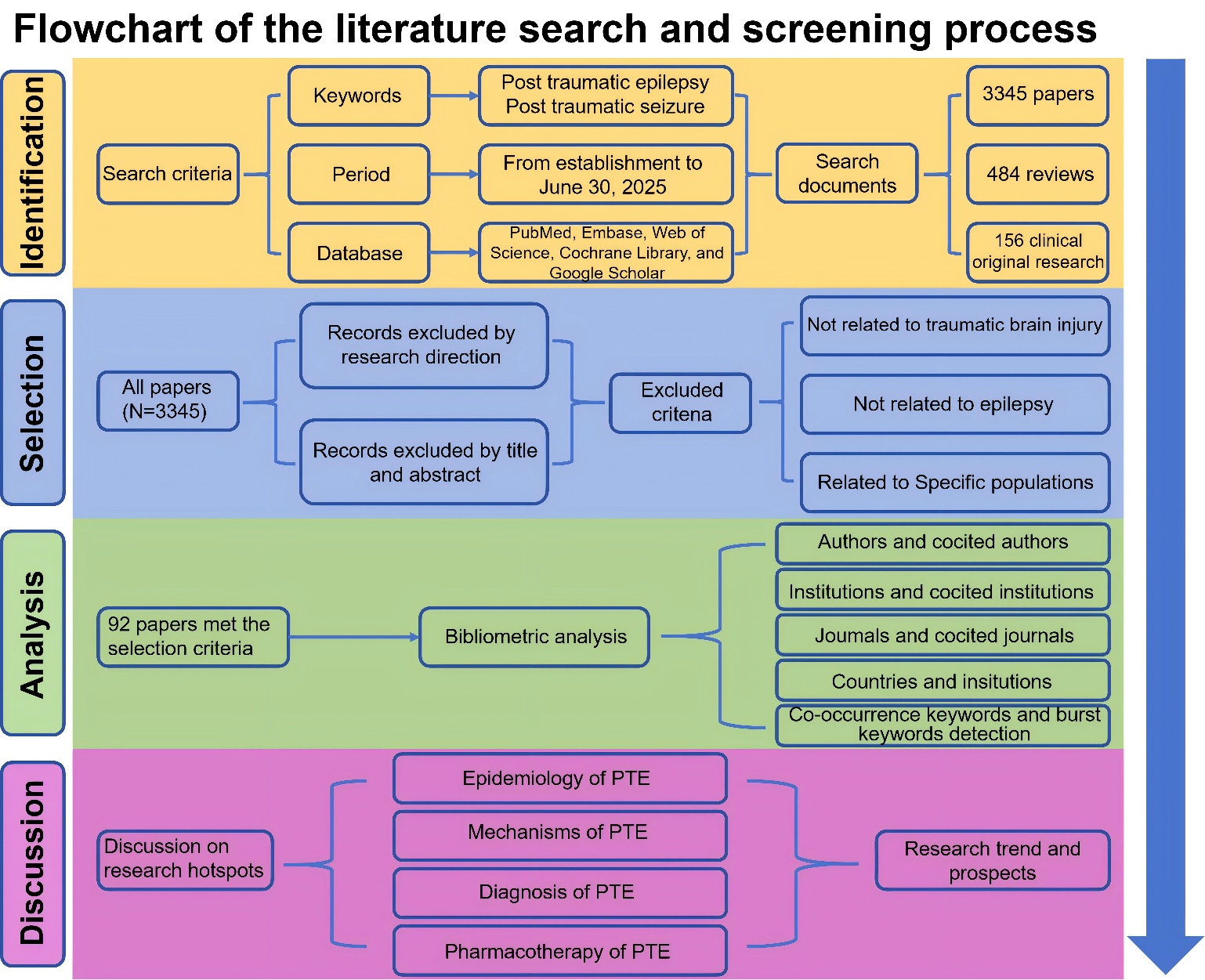
**

**Supplementary figure 1** Flow diagram for included/excluded studies
